# Raman imaging for the investigation of *Mycobacterium smegmatis* in a mother machine

**DOI:** 10.1007/s00216-025-06307-y

**Published:** 2026-01-14

**Authors:** Ida Kalleder, Eva Krois, Karin Wieland, Anna-Cathrine Neumann-Cip, Charlott Leu, Andreas Wieser, Susanna Oswald, Christoph Haisch

**Affiliations:** 1 Chair of Analytical Chemistry and Water Chemistry, Technical University of Munich, Munich, Germany; 2 Fraunhofer Institute for Translational Medicine and Pharmacology, Immunology, Infection and Pandemic Research, ITMP-IIP, Munich, Germany; 3 Competence Center Chase GmbH, Vienna, Austria; 4 Institute of Infectious Diseases and Tropical Medicine, Ludwig-Maximilians-University Hospital, Munich, Germany; 5 German Center for Infection Research (DZIF), Partner Site Munich, Munich, Germany; 6 Chair of Medical Microbiology and Hospital Epidemiology, Max Von Pettenkofer Institute, Faculty of Medicine, Ludwig-Maximilians-University, Munich, Germany; 7 Chair of Soft Matter Physics, Ludwig-Maximilians-University, Munich, Germany

**Keywords:** *M. smegmatis*, Mother machine, Raman spectroscopy, Microfluidics, Live cell imaging, Isotope labelling

## Abstract

The mother machine (MM) is a microfluidic device designed to trap and measure single bacteria for long-term experiments, enabling the study of bacterial growth and cell cycle dynamics. In this work, the concept of a MM is adapted to fit the requirements for investigating *Mycobacterium smegmatis*, a bacterium used extensively as a model system in tuberculosis research. A MM chip was fabricated using soft lithography, and a protocol for sample preparation and filling of the side channels for mycobacteria was established. Trapped bacteria were measured using Raman spectroscopy to introduce a new analysis approach inside the MM. The subsequent data evaluation demonstrates the potential for obtaining spatially resolved chemical information on a single cell within a side channel of the MM through Raman imaging. Furthermore, the combination of Raman imaging and stable isotope labelling, as applied in this study, demonstrates the viability of bacteria inside a side channel and opens up possibilities to study cell cycles in long-term experiments.

**Graphical abstract:**

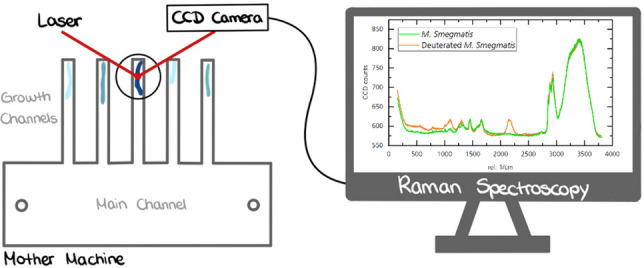

**Supplementary Information:**

The online version contains supplementary material available at 10.1007/s00216-025-06307-y.

## Introduction

The mother machine (MM) is a microfluidic lab-on-a-chip device that was first introduced by Wang et al. in 2010 [1]. It was constructed to enable long-term monitoring of single *Escherichia coli* (*E. coli*) bacteria. The name “mother machine” is derived from the so-called baby machine developed by Helmstetter et al. in the 1960s [2]. In the baby machine, cells are attached to a membrane, releasing “baby cells” through cell division. These generated cells can be washed away and used for subsequent investigation [2]. The MM enables trapping of a single bacterium in a dead-end channel and its observation under steady-state conditions [1]. In our study, the device is fabricated from polydimethylsiloxane (PDMS) using a soft lithography approach [3]. PDMS has the advantages of being transparent, biocompatible, inexpensive, and non-toxic [3, 4]. The miniaturization of the device reduces the sample volume and, consequently, the overall use of chemicals, resulting in a low-cost and easy-to-handle MM. The MM consists of a main channel with several hundred dead-end side channels perpendicular to it. The broader main channel ensures a constant nutrient supply through diffusion-based mass transport, whereas the high number of side channels enables a highly parallelized observation of the trapped bacteria. The dimensions of the side channels are chosen to fit the bacteria under investigation and enable the trapping of single bacteria while avoiding superpositioning. A bacterium that is trapped and permanently retained at the end of the side channel is defined as the “mother cell.” During each division, a new daughter cell of mycobacteria is generated. Unlike most bacteria, mycobacteria grow at their cell poles; thus, no old cell pole is expected to accumulate in the system as would be seen in *E. coli* [1, 5]. Still, bacterial cells originating from growth within each channel are pushed toward the main channel through continuous cell division and finally flushed away. This process keeps the colony’s size fixed, a prerequisite for the continuous observation of single cell progeny, and for upholding steady-state conditions [6]. Thus, the mother cell can be observed on a single-cell level over several cycles of cell division, enabling the investigation of aging mechanisms like cell heterogeneity or inheritance of aging factors [7–9].

The primary detection methods used in MMs are optical time-lapse microscopy [10, 11] and fluorescence microscopy [12]. Some groups focused on image analysis of recorded data by establishing automated image analysis software [11, 13, 14]. We introduce Raman spectroscopy, combined with a customized data evaluation pipeline, as an alternative detection method. Raman spectroscopy is a powerful tool for investigating bacteria on a single-cell level. It is based on Stokes scattering, an interaction between light and matter, usually initiated by a laser in the visible range as a monochromatic excitation light source. The interaction is characterized by an energy transfer between the incident photon and the molecule, resulting in the photon’s inelastic scattering. The difference in energy between the incident and detected light is characteristic of each molecule and provides chemical information about the sample in a label-free, non-destructive manner [15]. Furthermore, Raman imaging enables the mapping of single bacteria with subcellular resolution, delivering insights into their metabolism through the combination with stable isotope labelling.

Most MM studies focus on *E. coli* [16]. There are studies where other organisms have been introduced into the MM, such as *Salmonella* [17] or *Bacillus subtilis* [12]. To the best of our knowledge, no studies have been reported on introducing *Mycobacterium smegmatis* (*M. smegmatis*) into the MM, a model bacterium for *Mycobacterium tuberculosis* (*M. tuberculosis*), which causes tuberculosis, one of the deadliest infectious diseases worldwide [18]. Research on the bacteria’s metabolism and their response to environmental changes may provide valuable insights into new approaches for tuberculosis treatment.

This work tries to bridge the gap between the MM as a microfluidic device and Raman spectroscopy as a suitable tool for non-invasive single-cell studies. This is achieved by stable isotope labelling of *M. smegmatis* trapped inside the side channels of a customized MM for these bacteria.

## Materials and methods

### Fabrication of the Si master for the mother machine

The silicon master mold for the MM was fabricated in a multi-step photolithography process. In the first step, alignment marks were defined by direct laser lithography (LPKF Protolaser LDI) on an AZ MIR 701 (Merck KGaA, Darmstadt, Germany) resist-coated Si wafer, followed by deposition of a 100-nm chromium layer and subsequent lift-off, leaving well-defined Cr alignment marks. For the first device layer, a diluted (1:1 in cyclopentanone (Merck KGaA, Darmstadt, Germany)) SU-8 3010 (Micro Chem, Newton, USA) photoresist was spin-coated to achieve a thickness below 1 µm, enabling the definition of the narrow side channels (1 µm). After soft-bake, alignment to the chromium marks was performed, and the complete channel design (main channel and side channels) was exposed using the LDI system, developed in PGMEA (Merck KGaA, Darmstadt, Germany), and post-baked according to the datasheet. In the second layer, SU-8 50 (Micro Chem, Newton, USA) was spin-coated at 3000 rpm to achieve a thickness of 50 µm, defining the main supply channel. Again, alignment to the Cr marks, exposure with LDI, development in PGMEA (Merck KGaA, Darmstadt, Germany), and a final hard bake at 180 °C were carried out. Finally, the master surface was functionalized with trichloro(1H,1H,2H,2H-perfluorooctyl)silane (Sigma-Aldrich, St. Louis, USA) to facilitate later PDMS replication and demolding.

### Fabrication of the MM

The MM was produced by pouring freshly mixed and degassed PDMS (SYLGARD 184 Silicone Elastomer Kit, Dow Europe GMBH C/O Dow Silicones, Wiesbaden, Germany) in a 10:1 ratio (elastomer:cross-linker) over a Si-wafer master in a petri dish. After pouring the PDMS onto the Si-wafer master, the polymer mixture was degassed for 10 min. The petri dish was placed in an oven at 60 °C for 16 h. Afterward, the MM chips were cut out, and the holes for the connecting tubes were punched using a biopsy puncher with a diameter of 1.5 mm (Integra GmbH & Co KG, Rostock, Germany).

The cover glass (borosilicate glass slide, 24 × 60 mm, thickness no. 1 (0.13–0.16 mm) from Carl Roth, Karlsruhe, Germany) was cleaned twice for 5 min each in an ultrasonic bath using acetone, followed by isopropanol. Particle- and oil-free pressurized air was used to dry the cover glass. The cleaned glass slides and the PDMS MM masters were activated with sides later to be connected facing upwards, in the plasma cleaner (Harrick Plasma, Plasma Cleaner PDC-002-CE, NY, USA) for 2 min at 30 W. The activated sides of the PDMS and cover glass were immediately pressed together, and the chip was cured at 60 °C for 16 h. The inlet and outlet tubes with an inner diameter of 0.51 mm and an outer diameter of 1.52 mm (Tygon®, type: ND-100-80, S 54-HL) were cut into 20-cm-long pieces. The tubes and the MM chip were activated in the plasma cleaner prior to inserting the tubes into the tube holes.

### Preparation of *M. smegmatis*

*M. smegmatis* (mc^2^155) cultures were grown according to previous work [19] for 24 h in M7H9 medium (Middlebrook 7H9 Broth Base, Sigma-Aldrich, Buchs, Switzerland) supplemented with 10% oleic albumin dextrose catalase (BD, BBL™ Middlebrook OADC Enrichment, NJ, USA) and 0.05% Tween 80 (Sigma-Aldrich, Buchs, Switzerland) at 37 °C under constant shaking to reach a final optical density (OD_600_) of 1.0.

After incubation, the cell suspension was centrifuged (10,000 rcf; 10 min; 4 °C) and washed using 10 mL PBS. The cells were then concentrated by a factor of 20 through resuspension in PBS + 1% Tween 80 + 1% BSA (Albumin Fraktion V, NZ-Origin, Carl Roth, Karlsruhe, Germany).

### Protocol for the population of side channels with bacteria

A blunt cannula with a 0.8-mm diameter and a length of 22 mm (Sterican®, *B. Braun Petzold GmbH*, Melsungen, Germany) was mounted onto the inlet tubes of the channel. These were used to connect the channel to a syringe pump later on. The channel was first flushed with 2 mL of 1% BSA in H_2_O (Ampuwa®, Fresenius Kabi AG, Homburg, Germany) with a 100 µL/min flow rate to passivate the surface. Afterward, the chip was left at room temperature (RT) for 45 min. 0.2 mL of the bacteria suspension was flushed through the channel with a 50 µL/min flow rate. The chip was stored with the cover glass facing down at RT for 90 min. The inlets and outlets were covered with a water-soaked tissue to prevent the channel from drying out. An adapter was used to allow centrifugation (1200 rcf; 4 °C; 45 min) with the centrifugal forces acting in the direction of the side channels. Subsequently, the MM main channel was flushed with 1 mL of 1× PBS (flow rate of 100 µL/min) to remove excess bacteria.

For overnight experiments (16 h) with bacteria already inside side channels, the MM was incubated at 37 °C while constantly flushing with (deuterated) medium at a flow rate of 25 µL/min. After incubation, the channel was flushed again with 2 mL PBS (flow rate of 50 µL/min) before Raman spectroscopic measurements.

### Raman measurements

Raman spectra were collected with a WITec alpha300 R system (Oxford Instruments, Abingdon, Oxfordshire, UK (formerly WITec GmbH, Ulm, Germany)) equipped with a Cobolt DPL 532 nm solid-state laser (Cobolt AB, Solna, Sweden) and a TruePower module. The used objective was a Plan-APOCHROMAT 63× oil DIC objective (Zeiss C Plan-Apochromat 63 × 1.4 Oil DIC, Carl Zeiss AG, Oberkochen, Germany). A spectrometer grating with 600 lines/mm was used, and detection was performed with a Newton 970 EMCCD camera (Andor Technology Ltd., Belfast, UK). We acquired spectra of bacteria using a 15-mW laser power, an acquisition time of 1 s, and a single accumulation in the spectral range from 150 to 3800 cm^−1^. The spectral images were recorded with a step size of 230 nm.

### Data evaluation

The acquired data were evaluated using an in-house developed MatLab (MatLab R2023b) script, which includes the following steps: loading raw data and reference files for cover glass, buffer, PDMS, *M. smegmatis*, and deuterated *M. smegmatis*. Then, all data, including reference spectra, are processed using background subtraction [20], Savitzky-Golay smoother for cosmic ray removal (polynomial order, 3; frame length, 5), and a min-max normalization. A multilinear regression (MLR) was performed, covering the spectral range from 400 to 3500 cm^−1^. The regression components are the reference spectra for glass, buffer, PDMS, and *M. smegmatis*. False-color images were created based on the regression coefficients (*b*-values) from the MLR for PDMS, PBS, and biomass to investigate undeuterated bacteria and show their distribution across the large area scan. A reference spectrum of deuterated *M. smegmatis* was added as a fifth component for the evaluation of deuterated bacteria. To investigate the degree of deuteration inside a deuterated bacterium, a second MLR was performed in the spectral range between 1500 and 3100 cm^−1^ for PDMS, PBS, undeuterated *M. smegmatis*, and deuterated *M. smegmatis*. This second analysis was performed only for spectra with a positive *b*-value for undeuterated *M. smegmatis* in the first MLR [21]. The ratio of the *b*-values of the deuterated bacteria to the *b*-value of the undeuterated bacteria is depicted in false-color images. The MatLab script for data evaluation can be found in the supporting information.

## Results and discussion

### Design of the MM and population of its side channels with* M. smegmatis*

In the first step, a MM was designed to meet the requirements of *M. smegmatis*, and a protocol for trapping the bacteria within the side channels was established. Starting from existing MMs described in literature [1], which trapped *E. coli*, various dimensions of side channels were tested. *M. smegmatis* and *E. coli* are roughly similar in size, with a length of 3.0–5.0 µm for *M. smegmatis* and 2.0–6.0 µm for *E. coli* and a diameter of 0.6 µm and 1.1 µm, respectively [22, 23]. The key differences between the two kinds of bacteria are that *E. coli* are motile through peritrichous flagella, whereas *M. smegmatis* are non-motile. Furthermore, *E. coli* has a doubling time of around 20 min compared to a doubling time of 3 h for *M. smegmatis* [24, 25]. Additionally, the cell wall of mycobacteria is rigid and hydrophobic, whereas that of *E. coli* is more flexible and hydrophilic, which can impact experiments related to bacterial aggregation and adhesion [26]. Consequently, *M. smegmatis* are harder to handle inside a MM because firstly they cannot enter side channels through their own motility; secondly, they need longer experiment times due to longer doubling times when it comes to the observation of metabolism and aging inside side channels; and thirdly, the addition of a detergent to medium or buffer is necessary to avoid adhesion of the bacteria to channel walls or other bacteria.

**Fig. 1:**
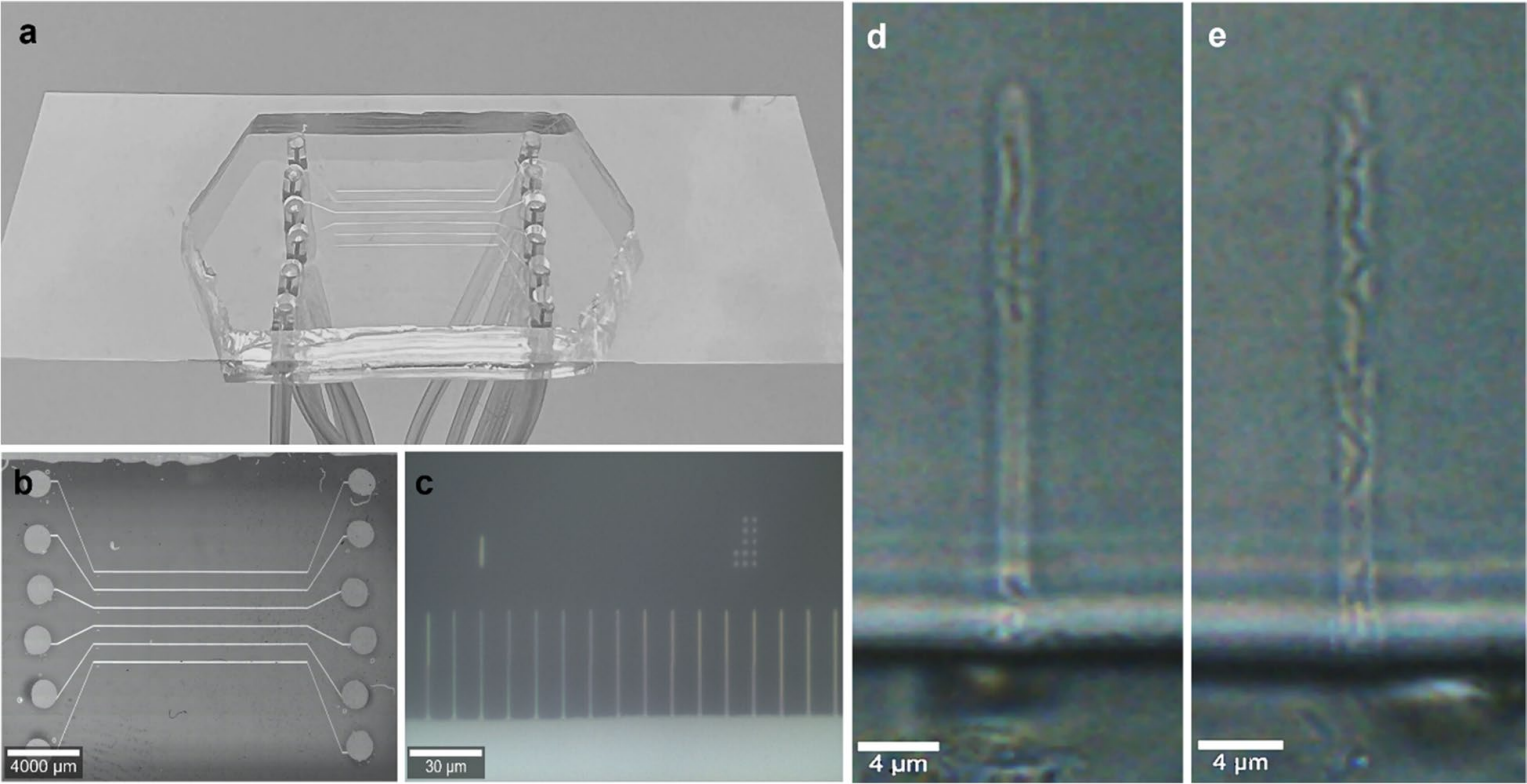
Structure of a MM: **a** picture of the MM, including inlet and outlet tubes; **b** microscopy image (5× magnification) showing the six main channels; **c** microscope image of 2-µm channels (63× magnification) with markings corresponding to the position along the main channel; **d** image of side channels after initial population (63× magnification); **e** image of the same side channel as shown in d after incubation overnight

The MM chip comprises six identical channel systems (Fig. 1a, b), each characterized by a 100.0-µm-wide main channel with a depth of 35.0 µm and a length of 9.0 mm. Side channels (1.0 µm depth, 2.0 µm width, 30.0 µm length) are oriented perpendicular to the main channel, with the upper edge of the main channel and the side channels at the same level. A glass slide (0.13–0.16 mm thick) is used to cover all main and side channels, allowing for Raman spectroscopy within the channels. An interspace of 10.0 µm between each side channel avoids the collapse of the side channels during centrifugation. As the MM is moved and handled during experiments, e.g., for incubation or centrifugation steps, it is crucial to implement markings to identify the exact same side channel with the respective bacterium under investigation after each cultivation/incubation step. Therefore, every 20th side channel is marked with the number of dots corresponding to its position divided by 20. A line marking is set in between to mark every 10th channel (see Fig. 1c).

The dimensions of the side channels with a depth of 1.0 µm, a width of 2.0 µm, and a length of 30.0 µm were adapted for optimal trapping of *M. smegmatis*. These dimensions enable a sufficient percentage of populated side channels, and simultaneously, the channels are narrow enough to avoid complete or partial superpositioning of bacteria. In theory, due to the dimensions of a single bacterium, a size of 1.0 µm × 1.0 µm of side channels should be sufficient to trap *M. smegmatis*. As these dimensions are close to the spatial resolution achieved with the photolithographic chip fabrication process of the master, the fabricated channels with these dimensions were of minor quality. Therefore, no trapping of *M. smegmatis* in 1.0-µm-wide side channels was achieved.

A centrifugation step was added to the preparation protocol to overcome the problem of *M. smegmatis*’ lack of mobility. Before centrifugation, the MM system was left standing upside down for 90 min, letting the bacteria settle down on the glass cover, i.e., the level of the side channels, thus facilitating the access of the bacteria into these. The population of side channels was checked visually with 50× magnification.

Figure 1d, e demonstrates that it is possible to populate side channels (width of 2 µm) with *M. smegmatis* using the above protocol. With the established protocol, 10 to 44% of side channels in the direction of centrifugation are populated. In 80% of these, the bacterium is located near the dead end of the side channel. These bacteria are defined as mother cells and can be observed and measured throughout their cell cycle. Next, the MM was incubated overnight under constant nutrient supply. Figure 1d and e show the same side channel before and after incubation overnight. The bacterium at the end of the channel on day 1 grew and split overnight in consecutive cycles, populating the entire channel. Given the length of a side channel (30.0 µm), the minimum length of a single *M. smegmatis* bacterium (3.0 µm), and an estimated doubling time of 3 h, the incubation overnight (16 h) is three times the necessary amount of bacteria to populate the whole side channel. These tests prove that a bacterium at the dead end of a side channel can still grow and divide. Hence, the established procedure not only allows for the trapping of bacteria but also enables the performance of controlled, long-term experiments, as bacteria remain viable in side channels and, based on the markings on the chip, can be easily located again in repeated experiments.

### Raman imaging as an analysis method for* M. smegmatis *metabolism and growth in side channels

**Fig. 2:**
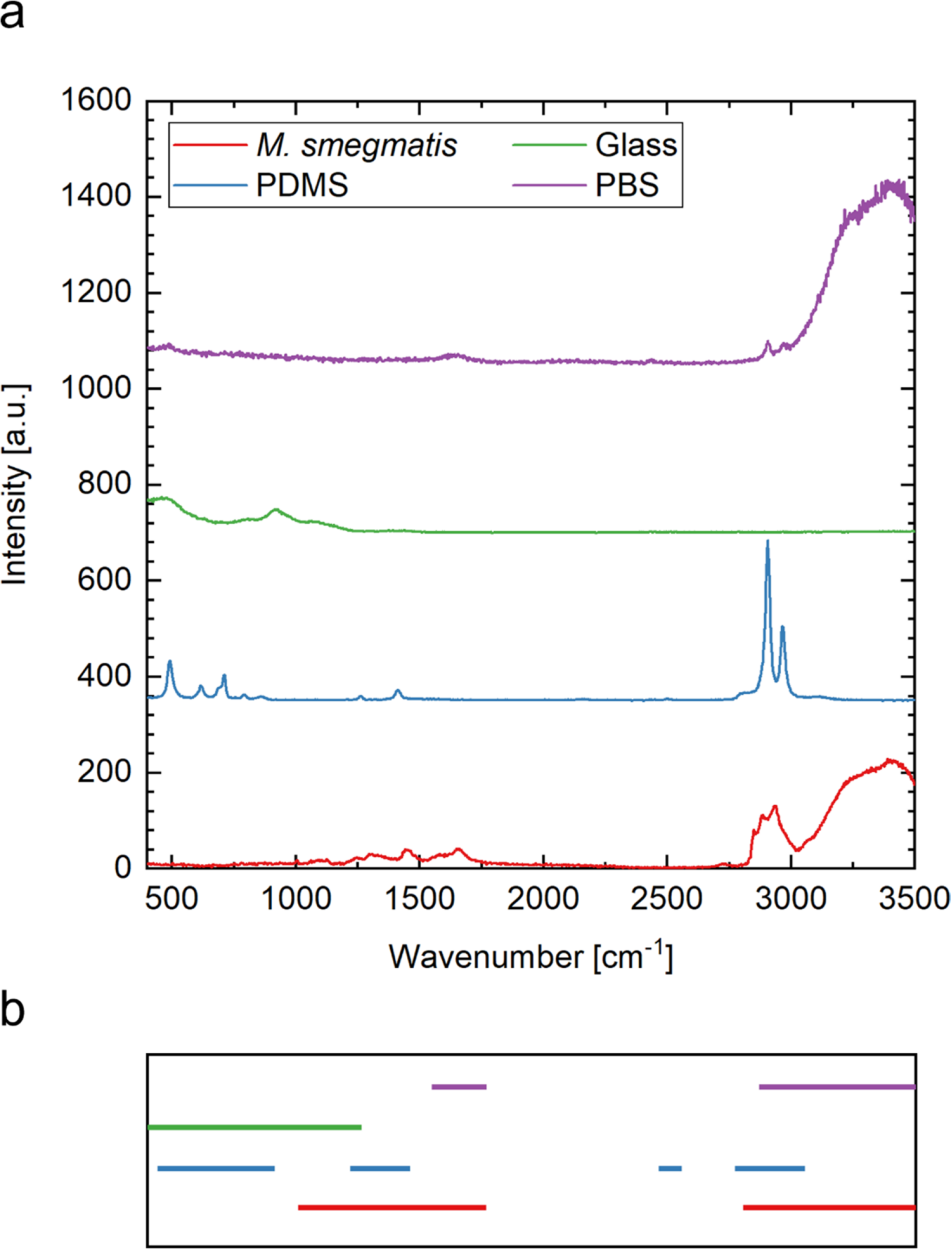
Reference Raman spectra (laser power = 15 mW, 1 s acquisition time, one accumulation) of PBS (violet), borosilicate glass (green), PDMS (blue), and *M*. *smegmatis* (red). **a** Raman spectra of references with an offset of 350 a.u. **b** Schematic depiction of spectral overlap of the different components (in the respective colors)

After successfully populating side channels with *M. smegmatis*, Raman measurements were performed. All Raman single spectra and images of living bacteria trapped inside the side channels were recorded through the glass cover of the MM. The Raman images indicate the presence of three other components in addition to the typical fingerprint of bacteria: PBS used as a buffer during experiments, PDMS, and glass. Figure 2a depicts reference spectra of all components taken in the MM to ensure the same conditions as in imaging experiments. Detailed band assignments can be found in Table S1 in the Supplementary Information.

Considering the transparent medium and the channel height of only 1 µm, the collected Raman spectra inevitably contain components from the bottom layers. PDMS is expected to contribute to all recorded spectra of any image taken inside the MM. Because PBS mainly consists of water, these bands are primarily seen in the corresponding spectrum. As all measurements are conducted through the cover glass, glass also contributes to the spectral fingerprint of the recorded Raman images.

**Fig. 3:**
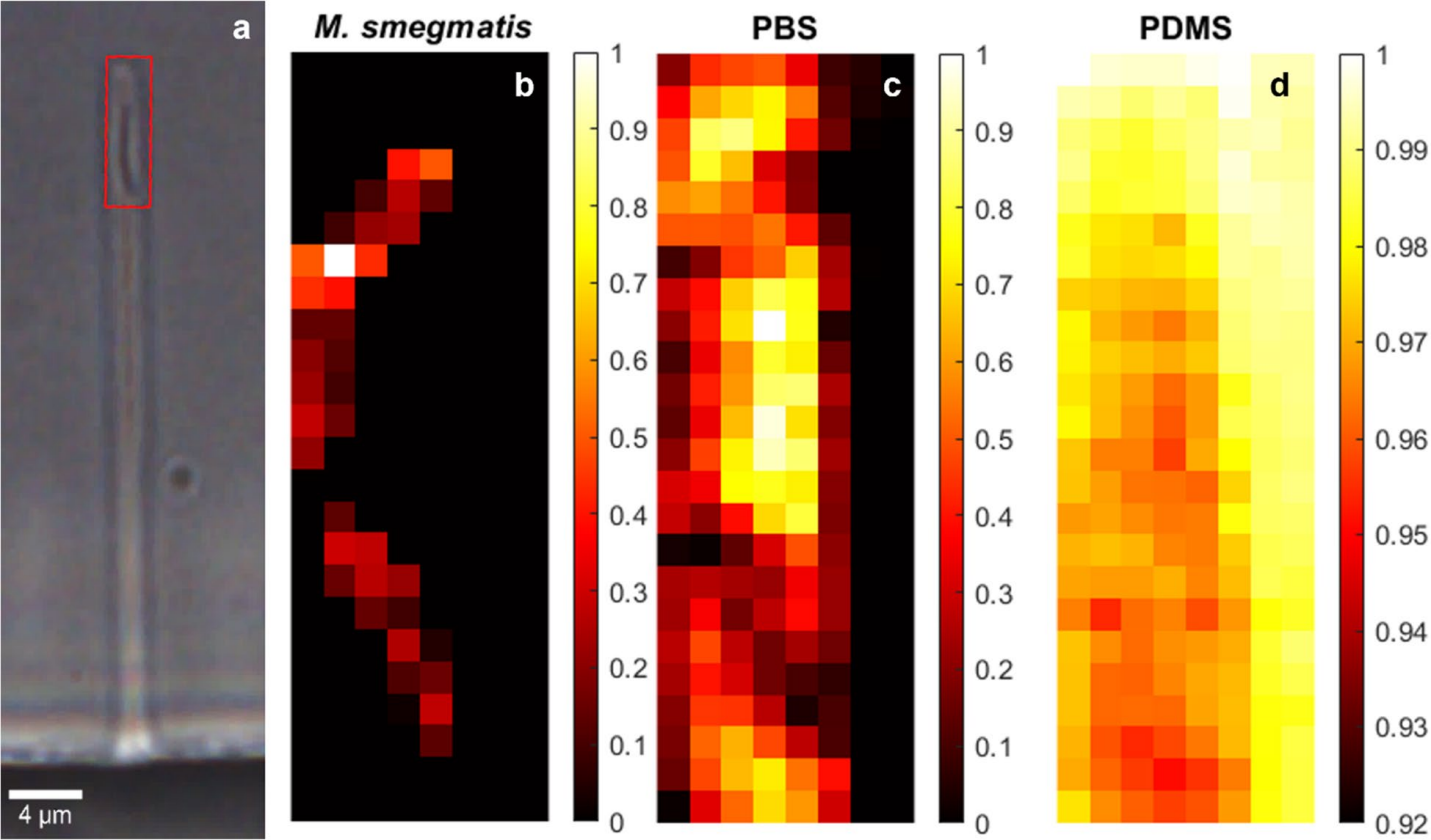
False-color Raman images of a single living *M. smegmatis* bacterium trapped in a side channel evaluated through multilinear regression. **a** Microscope image of a channel with the measured area marked by a red rectangle. **b** False-color image of the spatial distribution of *M. smegmatis*. **c** False-color image of the PBS distribution. **d** False-color image of the PDMS distribution in the measured area

Several of the pure component reference spectra overlap in the fingerprint region below 2000 cm^−1^ as well as in the CH/OH stretching region above 2700 cm^−1^ (see Fig. 2a, b). The CH stretching vibrations of PDMS and *M. smegmatis* overlap in the area above 2700 cm^−1^. Moreover, the OH stretching of water (2900–3700 cm^−1^) is visible in the spectra of bacteria as well as in the buffer. The amide I band of *M. smegmatis* at 1657 cm^−1^ overlaps with the HOH bending vibration of water at 1640 cm^−1^. Taking both factors into account, namely (i) the contribution of PDMS to all components inside a channel (blue line in Fig. 2b) and (ii) the overlap of *M. smegmatis* specific spectral features (red line in Fig. 2b) with those characteristic for glass (green line in Fig. 2b) and buffer (purple line in Fig. 2b), a simple approach like the integration of compound-specific bands would lead to an over- or underestimation of the individual compounds. Consequently, we employed an MLR for Raman image evaluation to unmix the identified components in the collected Raman spectra. This approach is based on the full spectra of the components PBS, glass, PDMS, and *M. smegmatis* (the complete script can be found in the SI). The regression coefficients (*b*-values) obtained for the different components are depicted in false-color images presented in Fig. 3.

The data evaluated by MLR reveal the lateral distribution of each compound (for more examples, see Figs. S1-S3). The pixels containing contributions of the characteristic *M. smegmatis* bands are presented in Fig. 3b. This structure is consistent with the light microscopic images. In contrast, the distribution of the buffer component in Fig. 3c indicates lower *b*-values for pixels where bacteria are present. A bacterium consists of considerable amounts of water (see Fig. 2), which explains that the buffer distribution is only lower and not zero in these areas. PDMS is the dominant component in each spectrum of the Raman image (see Fig. S4). Thus, the *b*-values of PDMS have a different scaling (0.92 to 1.0) to achieve a good contrast, revealing that the distribution of PDMS is consistent with the shape of the channel. This demonstrates that chemical information on the target organism is gained in a non-destructive way without fixation through Raman imaging, which opens up a broad range of opportunities for further experiments, e.g., by introducing tags or labels, such as stable isotope labelling, a commonly used technique in Raman spectroscopy to monitor metabolic activity of organisms. The employed MLR enables us to specifically demix spectra that show bacterial features from those that do not. This is an important prerequisite for identifying different features within a single cell, which will be described in the following section.

### Investigation of isotope-labelled bacteria in the MM

**Fig. 4:**
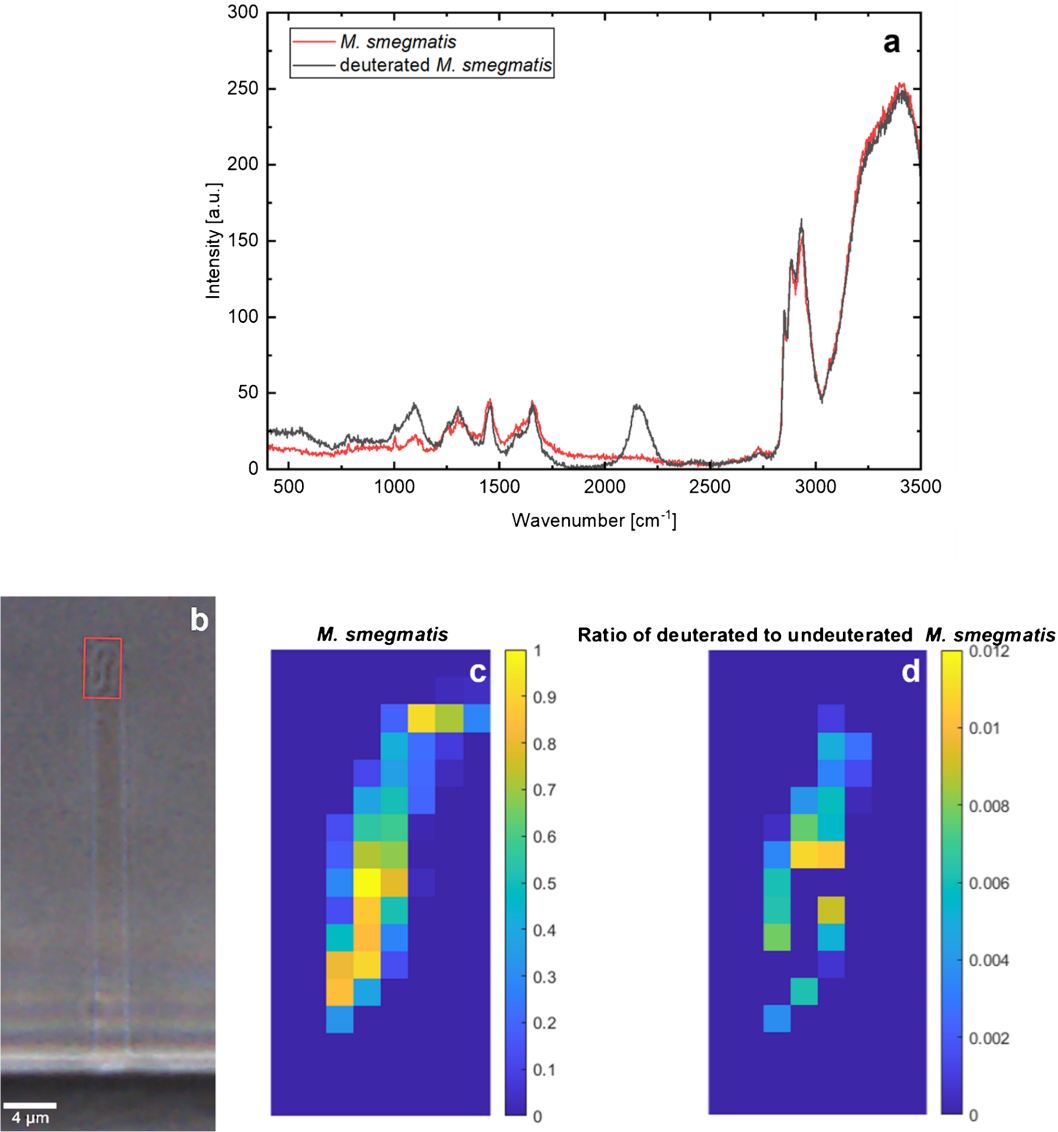
False-color images of deuterated *M. smegmatis* in a side channel. **a** Raman spectrum of deuterated and undeuterated *M*. *smegmatis*. **b** Microscope image of a 2-µm channel with the measured area indicated by a red rectangle. **c** False-color image of the distribution of undeuterated *M*. *smegmatis* (*b*-value). **d** Ratio of deuterated to undeuterated *M. smegmatis*

Stable-isotope labelling is an effective technique for the analysis of bacteria via Raman spectroscopy [21, 27]. We have recently discussed ^13^C labelling of Mycobacteria in combination with MALDI imaging [28]. Here, we demonstrate the possibilities of Raman spectroscopy in combination with deuterium labelling and the MM to provide further evidence for the viability and integrity of *M. smegmatis*. After bacteria were trapped inside channels, the buffer was changed to M7H9 containing 50% D_2_O, and the MM was incubated overnight at 37 °C at a constant flow rate of medium. After 16 h of incubation with deuterated medium, the medium was replaced by PBS, and Raman images were taken analogous to images of undeuterated bacteria. Only viable cells, which have an active metabolism, can take up the deuterated medium. The uptake leads to the appearance of a C-D stretching vibration in the spectrum and simultaneously to a decrease of the C-H stretching vibration. The C-D vibration is located at 2170 cm^−1^; a reference for deuterated *M. smegmatis* can be seen in Fig. 4a (see Figs. S5-S7 for more examples). The spectral range from 1800 to 2800 cm^−1^ is called the silent region, as it typically does not exhibit any spectral features in the analysis of biomolecules. Therefore, this region enables a very sensitive and robust detection of the C-D band free from spectral overlaps. However, in our MM system, there is significant spectral interference by the Si-H stretching vibration of PDMS [29] (see Fig. S8), originating from unreacted Si-H groups of the siloxane cross-linker. Thus, a second MLR between 1500 and 3100 cm^−1^ is conducted, including spectra of PDMS, PBS, as well as of deuterated and undeuterated *M. smegmatis*, for those spectra of the Raman image that feature a positive b-value for *M. smegmatis* in the first MLR. This pre-selection of spectra and the following limited spectral range eliminates glass as a reference, since glass has no bands in this spectral range, and also enables a more sensitive detection of the C-D band. The resulting *b*-values and *b*-value ratios are depicted in false-color images (Fig. 4c, d) showing the distribution of deuterated areas within *M. smegmatis*.

As can be appreciated in Fig. 4b, c, the position of the bacterium captured by brightfield microscopy shows good agreement with the false-color Raman image. The ratio of deuterated to undeuterated *M. smegmatis* (Fig. 4d) reveals the incorporation of deuterated medium either in the middle (yellow/orange pixels in Fig. 4d) or at the poles of the bacterium (Figs. S5-S7).

Thus, incorporation of the deuterated medium by the bacterium can be visualized. This result reproduces the expected phenotype from previous experiments in culture, where *M. smegmatis* could only incorporate deuterium as long as it was alive, underlining that the bacterium inside the side channel of the MM is still alive and has an active metabolism.

Furthermore, preliminary data suggest that bacteria with deuterium accumulation in the middle do not multiply, but are still metabolically active in the side channels, whereas for multiplying bacteria, deuterium accumulation is primarily observed at the poles (compare Figs. S5-S7), possibly highlighting different subpopulations of *M. smegmatis* within a culture.

To sum up, we are able to study the metabolism and growth of bacteria based on the chemical information gained by Raman spectroscopy. Isotope labelling is a valuable alternative to fluorescence-based live-dead staining, one of the state-of-the-art methods for investigating the viability of bacteria.

## Conclusion

This work presents the adaptation of the existing concept of the MM to *M. smegmatis*, including workflows for bacteria cultivation, successful channel population, and deuterium labelling. Thus, it joins other examples on how to customize the MM for specific kinds of bacteria. In addition, a non-destructive, easy-to-implement, fixation-free, and label-free approach is achieved by introducing Raman spectroscopy as the analytical method of choice for single bacteria trapped in the side channels. In contrast to other optical methods, such as time-lapse microscopy or fluorescence labelling, Raman imaging provides molecule-specific, chemical information on the bacteria of choice. Moreover, it enables the introduction of stable isotope labelling as an additional analysis method to investigate metabolic activity or long-term changes within single cells.

## Outlook

The adapted MM design presented here can be applied to other bacteria with dimensions similar to *M. smegmatis*, requiring only minor adjustments to the workflows for cultivation and population of side channels. Once this is achieved, the MM can be used to investigate diverse microbiological research questions, for instance by testing bacteria against substances that affect their metabolism, such as antibiotics or toxins. Hence, our next steps might include antibiotic susceptibility tests for *M. smegmatis*, a model organism for *M. tuberculosis*. In the long term, it will be possible to investigate the effect of antibiotics or combinations thereof on bacterial metabolism in real-time, in vivo, and at the single-cell level. Additionally, it is possible to combine different analytical methods, such as time-lapse microscopy (time-resolved information) and Raman imaging (spatially resolved chemical information), to gain complementary information while using the same experimental setup.

## Supplementary Information

Below is the link to the electronic supplementary material.

Supplementary file1 (PDF 1.69 MB)

## Data Availability

All relevant data are presented in the manuscript.
