# Supplementary material for: Raman imaging for the investigation of Mycobacterium smegmatis in a mother machine
Source: Anal Bioanal Chem. 2026 Jan 14;418(16):5171–80. doi: 10.1007/s00216-025-06307-y (PMC13424743; doi:10.1007/s00216-025-06307-y)
Supplement: Supplementary file 1 — Supplementary file1 (PDF 1.69 MB) [file 216_2025_6307_MOESM1_ESM.pdf]

Raman Imaging for the Investigation of *Mycobacterium smegmatis* in a Mother Machine

Ida Kalleder[1,2,†], Eva Krois[1,2,†], Karin Wieland[1,3], Anna Neumann-Cip[4], Charlott Leu[5], Andreas Wieser[2,4], Susanna Oswald[2], Christoph Haisch[1,‡,]

[1] Chair of Analytical Chemistry and Water Chemistry, Technical University of Munich, Munich, Germany

[2] Fraunhofer Institute for Translational Medicine and Pharmacology, Immunology, Infection and Pandemic Research, ITMP-IIP, Munich, Germany

[3] Competence Center Chase GmbH, Vienna, Austria

[4] Division of Infectious Diseases and Tropical Medicine, Ludwig-Maximilians-University, Munich, Germany

German Center for Infection Research (DZIF), Partner Site Munich, Munich, Germany

Chair of Medical Microbiology and Hospital Epidemiology, Max von Pettenkofer Institute, Faculty of Medicine, Ludwig-Maximilians-University, Munich, Germany

[5] Chair of Soft Matter Physics, Ludwig-Maximilians-University, Munich, Germany

[†] These authors have contributed equally to this work and share first authorship

[‡] Correspondence author, contact: haisch@tum.de

Supporting Information

Table S1: Band assignment of Borosilicate glass, PDMS, PBS, and M. smegmatis.

**Vibration mode**	**Raman shift /cm^{-1}**
**PBS[1]**	
H-O-H bending	1640
O-H stretching	2900-3700

**Borosilicate glass[2, 3]**	
SiO_4 tetrahedra symmetrical stretching (four bridging oxygens)	464
Boroxol group	806
SiO_4 tetrahedra stretching (two non-bridging oxygens)	920
SiO_4 tetrahedra stretching (one non-bridging oxygen)	1074
BO_3 triangle vibration (one non-bridging oxygen)	1417

**PDMS[4]**	
Si-O-Si stretching	489
C-Si stretching	618
Si-C asymmetric stretching	707
CH_3 symmetric rocking	790
CH_3 symmetric rocking	861
CH_3 symmetric bending	1260
CH_3 asymmetric bending	1415
Si-H stretching	2160
CH_3 symmetric stretching	2904
CH_3 asymmetric stretching	2965

***M. smegmatis*[5-7]**	
Proteins, phenylalanine (C-C)	1004
Protein, lipids, carbohydrates (C-C, C-N, C-O)	1127
RNA	1240
CH_2 twisting	1301
Lipids, fatty acids, proteins (CH_2 and CH_3 deformation)	1457
Lipids (C=C stretching), proteins (Amide I)	1655

CH_2 stretching	2852
CH_2 asymmetric stretching	2880
CH_3 symmetric stretching	2935

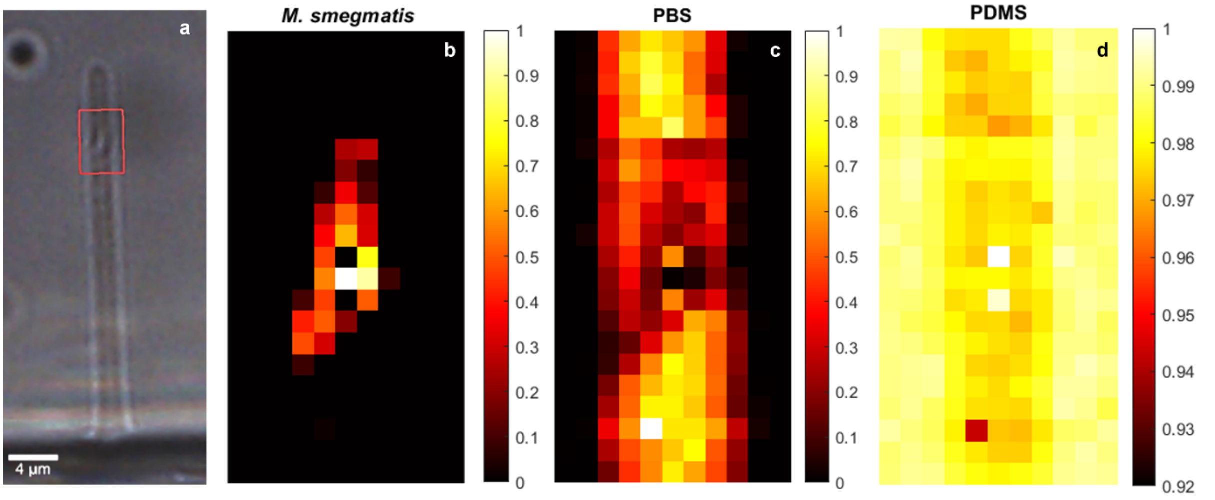

Fig S1 *False-color Raman images of a single living* M. smegmatis *bacterium trapped in a side channel evaluated through multilinear regression. a: Microscope image of a channel with the measured area marked by a red rectangle. b: False-color image of the spatial distribution of* M. smegmatis*; c: False-color image of the PBS distribution; and d: False-color image of the PDMS distribution in the measured area*

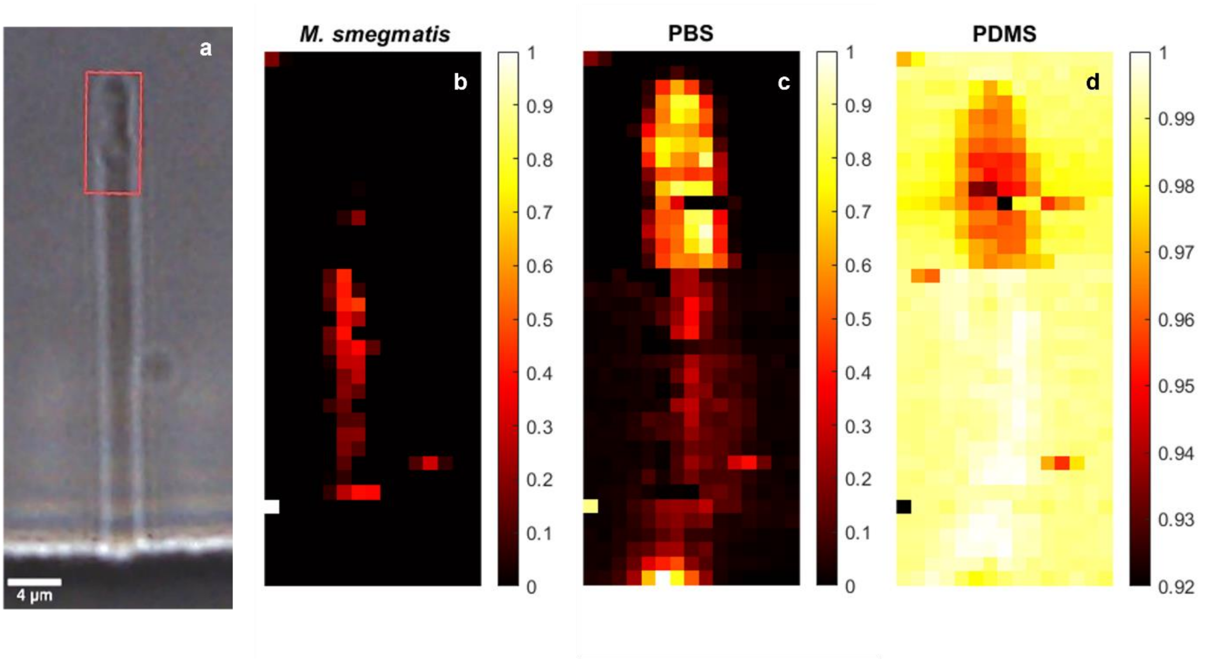

Fig S2 *False-color Raman images of a single living* M. smegmatis *bacterium trapped in a side channel evaluated through multilinear regression. a: Microscope image of a channel with the measured area marked by a red rectangle. b: False-color image of the spatial distribution of* M. smegmatis*; c: False-color image of the PBS distribution; and d: False-color image of the PDMS distribution in the measured area*

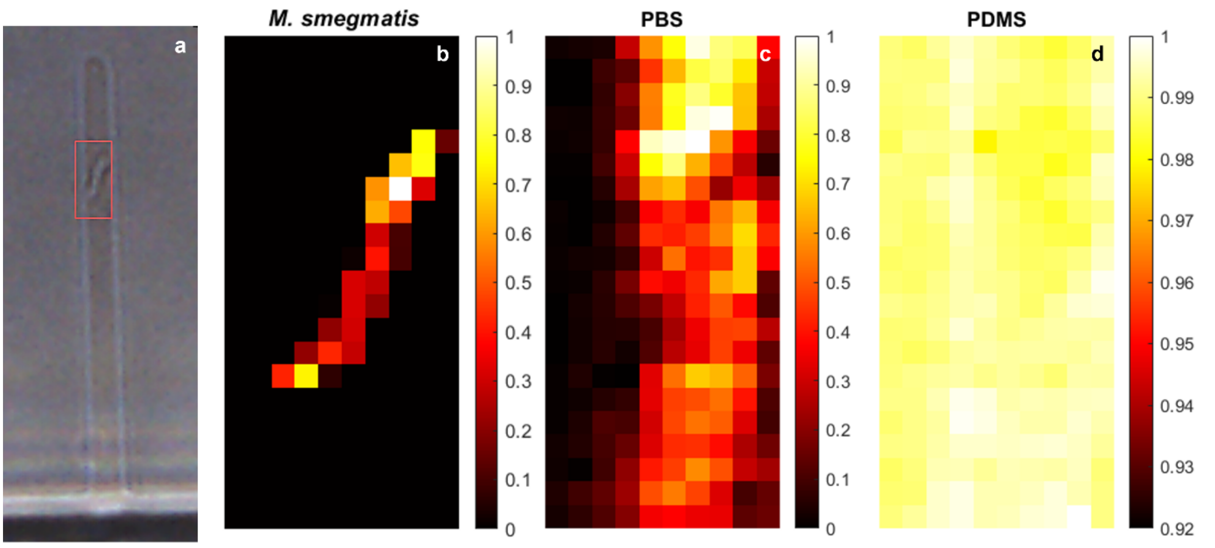

Fige S3 *False-color Raman images of a single living* M. smegmatis *bacterium trapped in a side channel evaluated through multilinear regression. a: Microscope image of a channel with the measured area marked by a red rectangle. b: False-color image of the spatial distribution of* M. smegmatis*; c: False-color image of the PBS distribution; and d: False-color image of the PDMS distribution in the measured area*

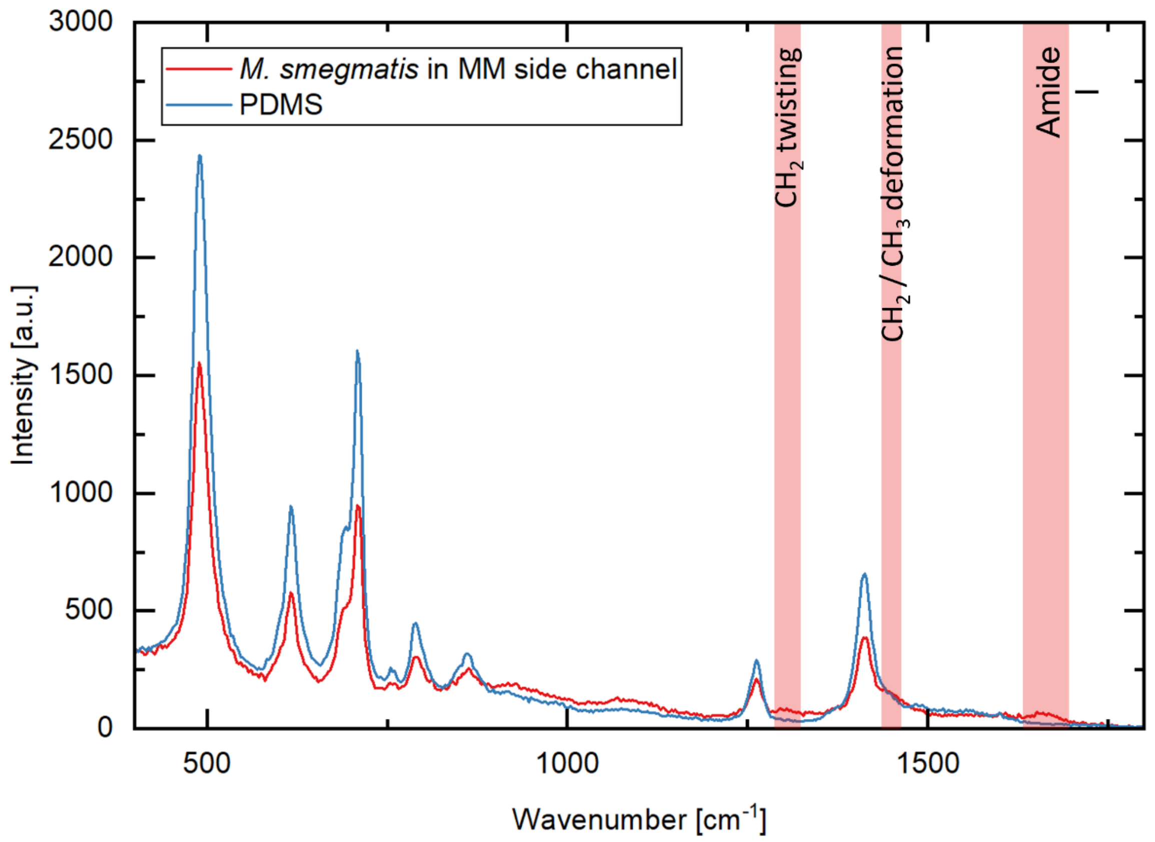

Fig S4 *Fingerprint area of a measurement of* M. smegmatis *(red) taken in a side channel of the MM and PDMS (blue) taken next to the side channel; red rectangles indicate areas with bacterial features in the spectrum dominated by PDMS.*

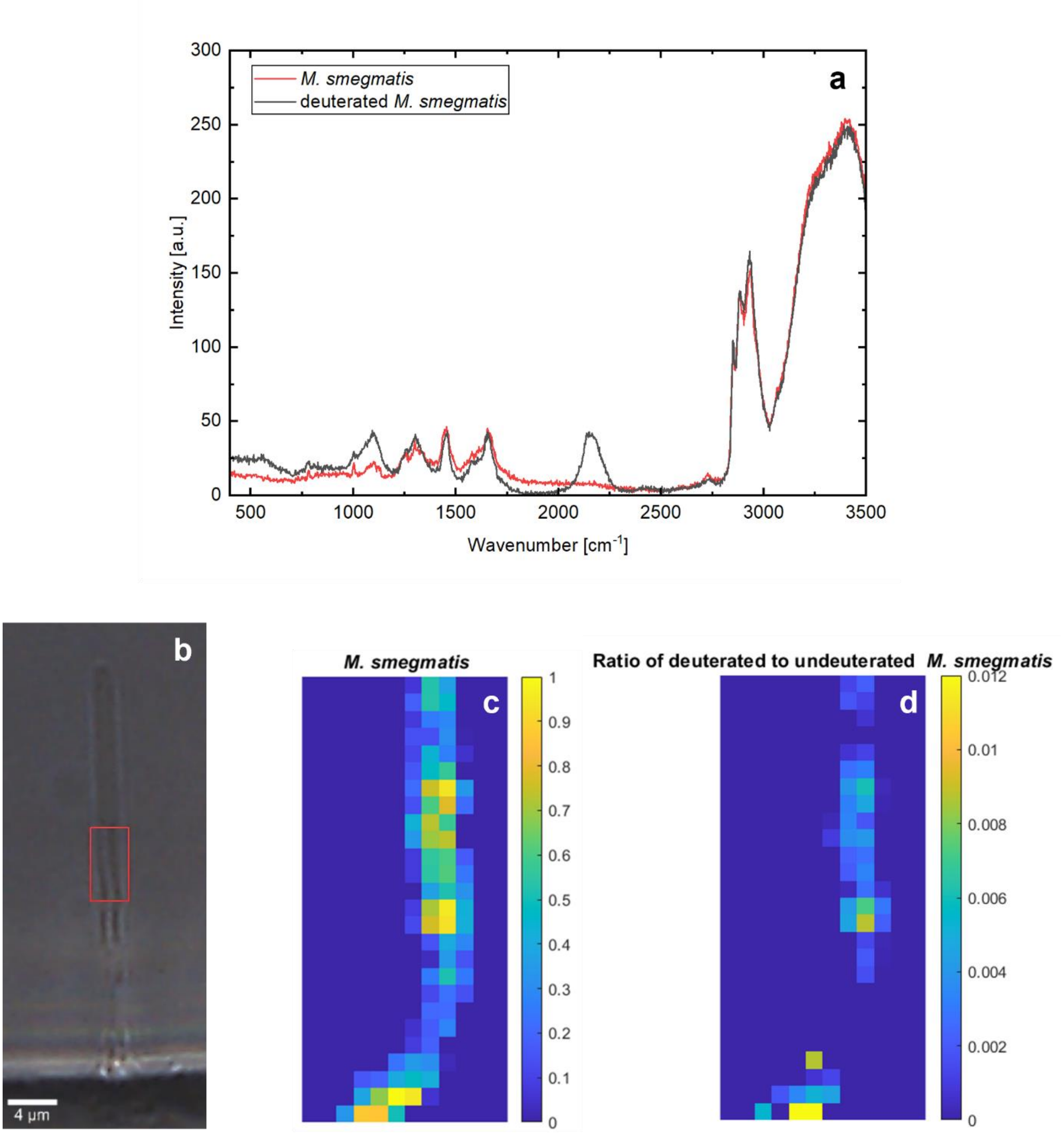

Fig S5 *False-color images of deuterated* M. smegmatis *in a side channel. a: Raman spectrum of deuterated and undeuterated* M. smegmatis; *b: Microscope image of a 2 µm channel with the measured area indicated by a red rectangle; c: False-color image of the distribution of undeuterated* M. smegmatis*; d: Ratio of deuterated to undeuterated* M. smegmatis

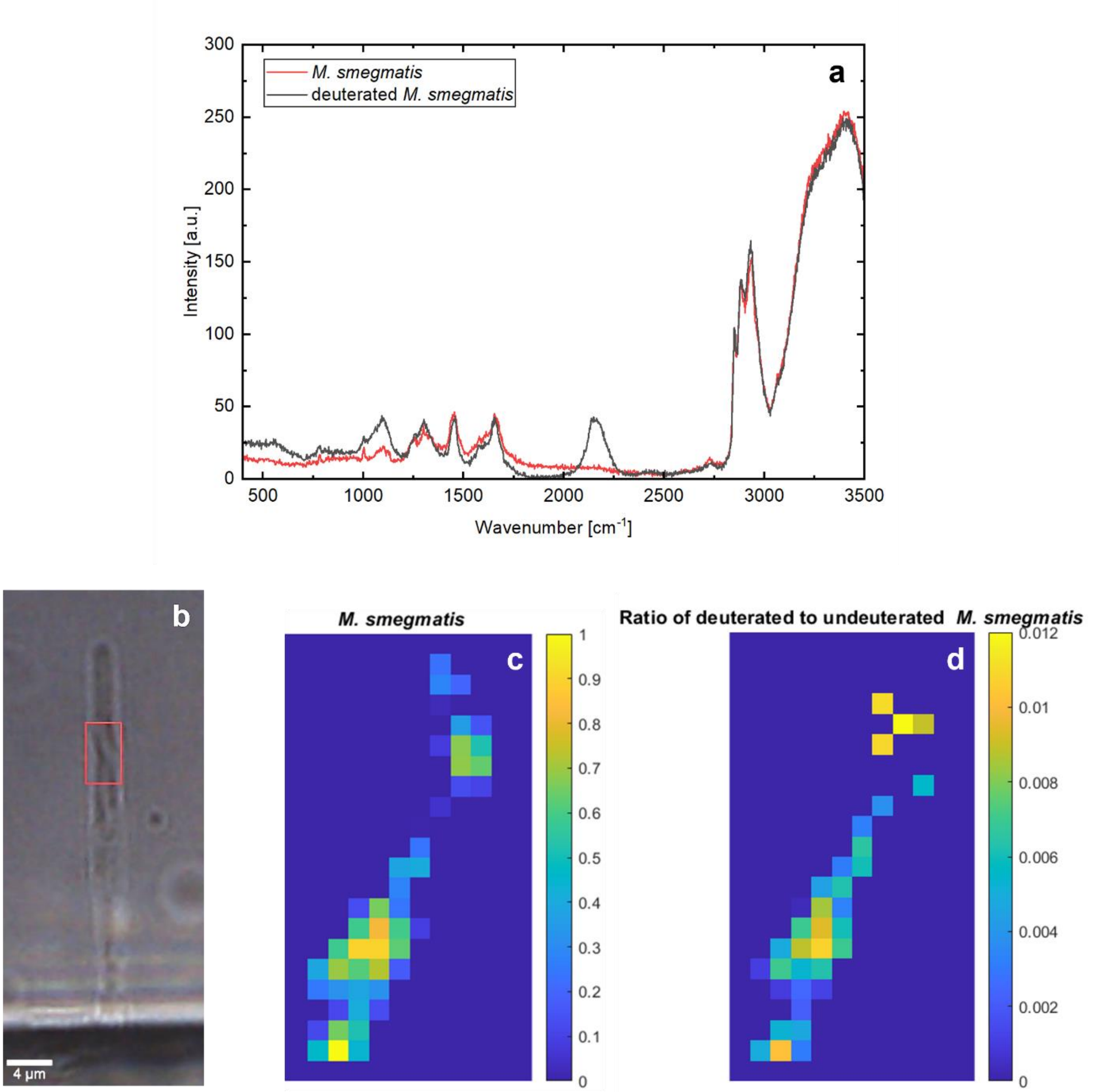

Fig S6 *False-color images of deuterated* M. smegmatis *in a side channel. a: Raman spectrum of deuterated and undeuterated* M. smegmatis; *b: Microscope image of a 2 µm channel with the measured area indicated by a red rectangle; c: False-color image of the distribution of undeuterated* M. smegmatis*; d: Ratio of deuterated to undeuterated* M. smegmatis

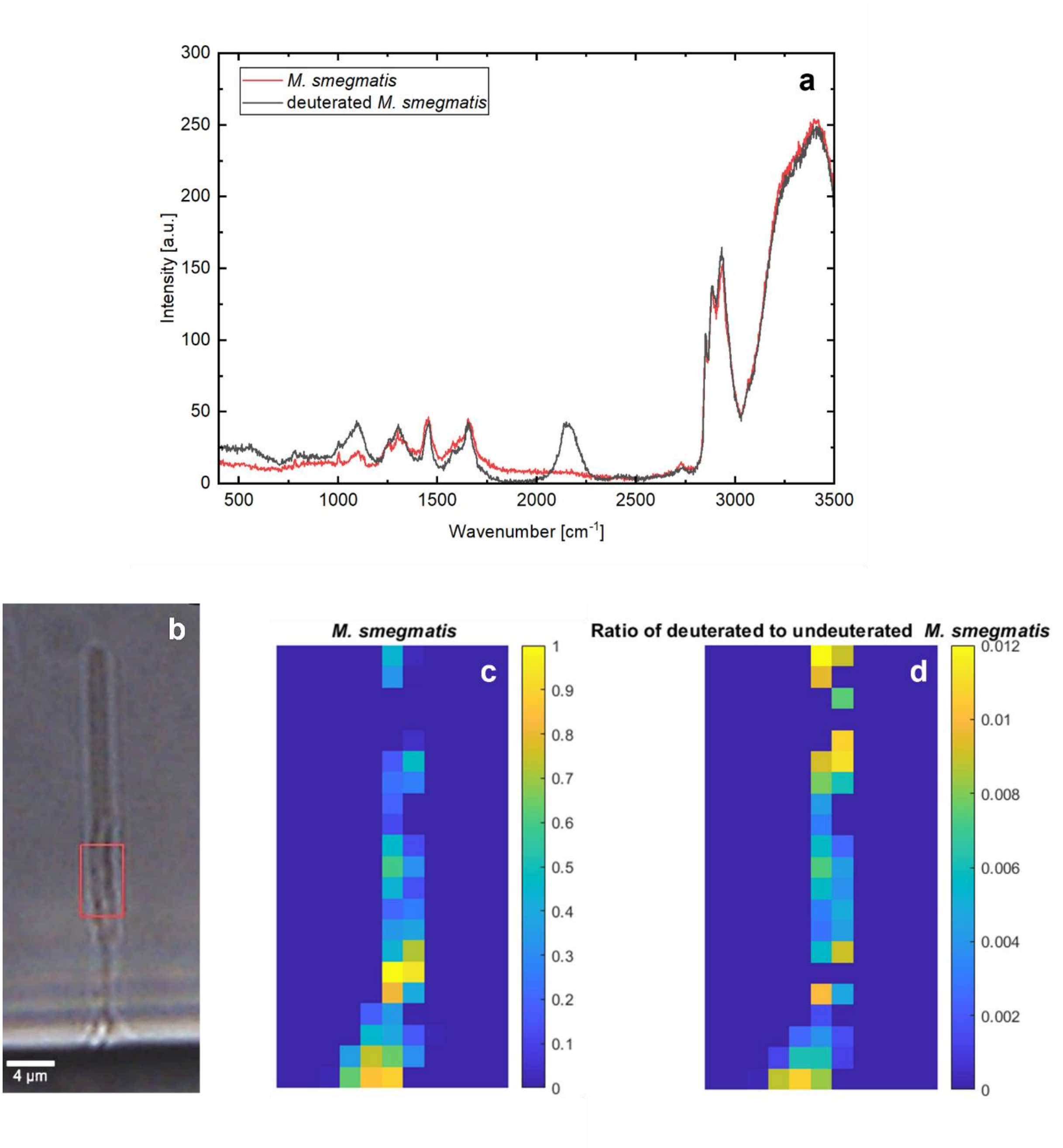

Fig S7 *False-color images of deuterated* M. smegmatis *in a side channel. a: Raman spectrum of deuterated and undeuterated* M. smegmatis; *b: Microscope image of a 2 µm channel with the measured area indicated by a red rectangle; c: False-color image of the distribution of undeuterated* M. smegmatis*; d: Ratio of deuterated to undeuterated* M. smegmatis

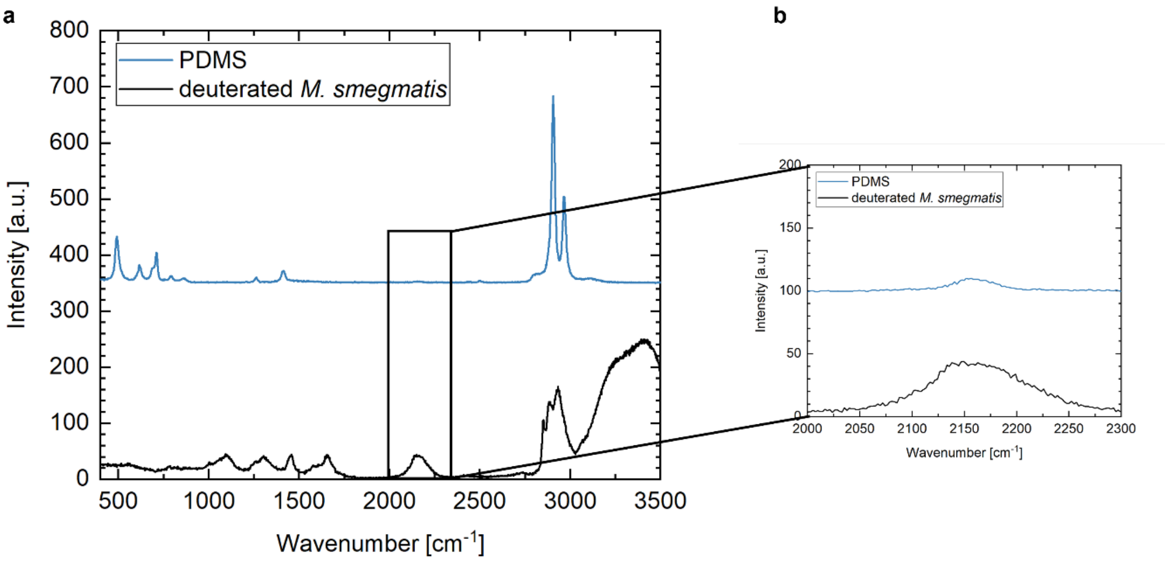

Fig S8 *Raman spectrum of PDMS (blue) and deuterated* M. smegmatis *(black); a: spectral area ranging from 400 to 3500 cm*$^{-1}$*, the rectangle marks the area zoomed in b, PDMS is offset by 350 a.u.; b: spectral area from 2000 to 2300 cm*$^{-1}$ *to demonstrate overlap of SiH stretching (PDMS) and CD stretching (*M. smegmatis*), spectra are shown with an offset of 100 a.u.*

Code for data evaluation

MatLab Code written in MATLAB R2024b. The first code is for the evaluation of Large Area Scans acquired for undeuterated bacteria, and the second code is for the evaluation of Large Area Scans acquired for deuterated bacteria in the Mother Machine.

Data processing for undeuterated bacteria.

```
%% Preprocessing and multilinear regression for WiTEC Raman images of undeuterated
bacteria

% Author: Felix Ludwig, felix.ludwig@tum.de
% Author: Ida. Kalleder, ida.kalleder@itmp.fraunhofer.de
% 06.08.2025

% simple .mat File loader for WiTec Image files with window selection,
% background correction, Min-Max-Normalisation and
% multilinreg with given References

% search for ….. to find all lines in which you have to add your personal pathway

clear all; % removing workspace artifacts
clc


%% Options
```

```
SavGolOrder = 3; % Oder of the Savitzky-Golay Filter Polynom
SavGolWindow = 5; % Window of the Savitzky-Golay Filter
minLimitWN = 400; % Lower Wavenumber Limit first Multilinear Regression
maxLimitWN = 3500; % Upper Wavenumber Limit first Multilinear Regression
minLimitWN2 = 1500; % Lower Wavenumber Limit second Multilinear Regression
maxLimitWN2 = 3100; % Upper Wavenumber Limit second Multilinear Regression


%% select file(s)

% Choosing the .mat data file
% Criteria: - .mat file
%           - image from WiTec (Project FIVE or similiar)

fBas = 'C:\.....'; % Paste in your file pathway!
[fileName, pathName] = uigetfile([fBas+"\"+'*.mat'], '*.mat',... % opens a
dialogue within the selected folder that have a .mat ending
'MultiSelect', 'on'); % *.mat : show only .mat files; MultiSelect enables to open
multiple .mat files at once

fprintf(" \n Loading raw data \n "); % gives you written output
tic % times how long is takes

fullPath = fullfile(pathName, fileName); % gives you the full path to your data as
output

rawImage.fileName = fileName;
rawImage.fullPath = fullPath;


%% Load File

% As the name implies. Raw data and information will be stored in rawImage.

image = load(fullPath,"-mat");  %the selected folder is loaded
image = struct2cell(image(1,1)); % converts a structure into a cell array
rawImage.Data = image{1,1}.data; % displays the data in the array as an image
rawImage.Name = image{1,1}.name; % extracts name
rawImage.Date = image{1,1}.date; % extracts date
rawImage.Imagesize = image{1,1}.imagesize; % extracts imagesize
rawImage.Axisscale = image{1,1}.axisscale; % extracts axis scale
rawImage.Imageaxisscale = image{1,1}.imageaxisscale; % extracts image axis scale
imageSize = rawImage.Imagesize;

clear fileName;
clear fullPath;
clear pathName;
clear fBas;


%% Load References

% Loading predefined References and creating a Ref-matrix

load("C:\....."); % add the path to your glass reference
load("C:\....."); % add the path to your PBS reference
load("C:\....."); % add the path to your PDMS reference
load("C:\....."); % add the path to your undeuterated bacteria reference

rawRef(1,:) = zeros(1600,1);
rawRef(1,:) = glas.data; % glass
```

```
rawRef(2,:) = medium.data; % PBS
rawRef(3,:) = pdms.data; % PDMS
rawRef(4,:) = smeggi_undeuteriert.data; % undeuterated bacteria

toc
fprintf(" \n Now cutting raw data \n ");


%% Cutting

% Cutting WN-range to spec. Choose Limits in "Options".

[max, maxIndexWN] = min(abs((image{1,1}.axisscale{2,1}(:))- maxLimitWN));
[min, minIndexWN] = min(abs((image{1,1}.axisscale{2,1}(:))- minLimitWN));
xraw=(rawImage.Axisscale{2,1}(:));
clear image;

data_processing.processingVar.minLimitWN = minLimitWN;
data_processing.processingVar.maxLimitWN = maxLimitWN;
data_processing.processingVar.minIndexWN = minIndexWN;
data_processing.processingVar.maxIndexWN = maxIndexWN;
data_processing.processingVar.min = min;
data_processing.processingVar.max = max;

cutRef = rawRef(:,minIndexWN:maxIndexWN)';
cutData = double(rawImage.Data(:,minIndexWN:maxIndexWN)');


clear min;
clear max;
clear minLimitWN;
clear maxLimitWN;
clear minIndexWN;
clear maxIndexWN;

toc
fprintf(" \n Already preprocessing data \n ");


%% Data processing

% fist applying a background subtraction, based off Schulz et al. 2012
(https://doi.org/10.1366/11-06550)
% second applying a Savitzky-Golay smoother to remove cosmic ray signals,
% last, normalising the data via min-max to avoid z drift differences.

for n = 1:size(cutData,2)
    [Base, corr_cutData(:,n)] = baseline(cutData(:,n));
    corr_cut_SG_Data(:,n) = sgolayfilt(double(corr_cutData(:,n)), SavGolOrder,
SavGolWindow);
    corr_cut_SG_MinMax_Data(:,n) = (corr_cut_SG_Data(:,n) -
min(corr_cut_SG_Data(:,n))) / (max(corr_cut_SG_Data(:,n)) -
min(corr_cut_SG_Data(:,n)));
    range = max(corr_cut_SG_Data(:,n)) - min(corr_cut_SG_Data(:,n));
if range > 0
    corr_cut_SG_MinMax_Data(:,n) = (corr_cut_SG_Data(:,n) -
min(corr_cut_SG_Data(:,n))) / range;
else
    corr_cut_SG_MinMax_Data(:,n) = zeros(size(corr_cut_SG_Data(:,n)));
end
end
```

```
data_processing.Base  = Base;
data_processing.cutData  = cutData;
data_processing.corr_cutData  = corr_cutData;
data_processing.corr_cut_SG_Data  = corr_cut_SG_Data;
data_processing.corr_cut_SG_MinMax_Data  = corr_cut_SG_MinMax_Data;
processedData = corr_cut_SG_MinMax_Data;

clear n;
clear Base;
clear corr_cut_SG_MinMax_Data;
clear corr_cutData;
clear corr_cut_SG_Data;

toc
fprintf(" \n ...preprocessing reference data \n ");


%% Reference processing

% fist applying a background subtraction, based off Schulz et al. 2012
(https://doi.org/10.1366/11-06550)
% second applying a Savitzky-Golay smoother to remove cosmic ray signals,
% last, normalising the data via min-max to avoid z drift differences.

for n = 1:size(cutRef,2)
    [RefBase, corr_cutRef(:,n)] = baseline(cutRef(:,n));
end
corr_cut_SG_Ref = zeros(size(corr_cutRef));
for n = 1:size(corr_cutRef,2)
    corr_cut_SG_Ref(:,n) = sgolayfilt(double(corr_cutRef(:,n)), SavGolOrder,
SavGolWindow);
    corr_cut_SG_MinMax_Ref(:,n) = (corr_cut_SG_Ref(:,n) -
min(corr_cut_SG_Ref(:,n))) / (max(corr_cut_SG_Ref(:,n)) -
min(corr_cut_SG_Ref(:,n)));
end

data_processing.RefBase  = RefBase;
data_processing.cutRef  = cutRef;
data_processing.corr_cutRef  = corr_cutRef;
data_processing.corr_cut_SG_Ref  = corr_cut_SG_Ref;
data_processing.corr_cut_SG_MinMax_Ref  = corr_cut_SG_MinMax_Ref;

processedRef = corr_cut_SG_MinMax_Ref;

clear n;
clear RefBase;
clear corr_cutRef;
clear corr_cut_SG_Ref;
clear corr_cut_SG_MinMax_Ref;
clear cutData;
clear cutRef;

toc
fprintf( "\n finally calculating multilinear regression \n ");


%% Multilinear regression

% bog-standard multilinear regression
% b (coefficient vector) and r (residual matrix) are stored under regResults
```

```matlab
for n = 1:size(processedData,2)

    [b,bint,r] = regress(processedData(:,n),processedRef);
    regResults.b(:,n) = b;
    regResults.bint(n).bint = bint;
    regResults.r(:,n) = r;
end

sum_b = sum(regResults.b(:, :), 1);
b_norm = regResults.b ./ sum_b;
image_undeuteriert_norm = reshape(b_norm(4, :), rawImage.Imagesize);


%% CleanUp

clear n;
clear r;
clear bint;
toc
fprintf(" \n ...time for a coffee! \n ");


%% Visualization of the distribution of undeuterated bacteria

b_norm = regResults.b ./ sum(regResults.b, 1);

image_smeggi_undeuteriert = reshape(b_norm(4, :), rawImage.Imagesize);
image_smeggi_undeuteriert(image_smeggi_undeuteriert < 0) = 0;

if max(image_smeggi_undeuteriert(:)) > 0
    image_smeggi_undeuteriert = image_smeggi_undeuteriert /
max(image_smeggi_undeuteriert(:));
end

% Plot
figure;
imagesc(image_smeggi_undeuteriert);
axis image off;
colorbar;
title('\it M. smegmatis');
colormap(parula);

xticks = 1:round(imageSize(2)/5):imageSize(2); % z.B. 5 Ticks auf x-Achse
yticks = 1:round(imageSize(1)/5):imageSize(1); % z.B. 5 Ticks auf y-Achse
set(gca, 'XTick', xticks, 'YTick', yticks);
xlabel('Pixel / Spektrum (Spalten)');
ylabel('Pixel / Spektrum (Zeilen)');


%% Visualization of PDMS distribution

image_pdms = reshape(b_norm(3, :), rawImage.Imagesize);
image_pdms(image_pdms < 0) = 0;

if max(image_pdms(:)) > 0
    image_pdms = image_pdms / max(image_pdms(:));
end

% Plot
figure;
```

```
imagesc(image_pdms);
axis image off;
colorbar;
title('PDMS');
colormap(parula);

xticks = 1:round(imageSize(2)/5):imageSize(2);
yticks = 1:round(imageSize(1)/5):imageSize(1);
set(gca, 'XTick', xticks, 'YTick', yticks);
xlabel('Pixel / Spektrum (Spalten)');
ylabel('Pixel / Spektrum (Zeilen)');


%% Visualization of Medium (PBS) distribution

image_medium = reshape(b_norm(2, :), rawImage.Imagesize);
image_medium(image_medium < 0) = 0;

if max(image_medium(:)) > 0
    image_medium = image_medium / max(image_medium(:));
end

% Plot
figure;
imagesc(image_medium);
axis image off;
colorbar;
title('PBS');
colormap(parula);

xticks = 1:round(imageSize(2)/5):imageSize(2);
yticks = 1:round(imageSize(1)/5):imageSize(1);
set(gca, 'XTick', xticks, 'YTick', yticks);
xlabel('Pixel / Spektrum (Spalten)');
ylabel('Pixel / Spektrum (Zeilen)');
```

Data processing for deuterated bacteria.

```
%% Preprocessing and multilinear regression for WiTEC Raman images of deuterated
bacteria

% Author: Felix Ludwig, felix.ludwig@tum.de
% Author: Ida. Kalleder, ida.kalleder@itmp.fraunhofer.de
% 06.08.2025

% simple .mat File loader for WiTec Image files with window selection,
% background correction, Min-Max-Normalisation and
% multilinreg with given References

% search for ..... to find all lines in which you have to add your personal
pathway

clear all; % removing workspace artifacts
clc


%% Options

SavGolOrder = 3; % Oder of the Savitzky-Golay Filter Polynom
SavGolWindow = 5; % Window of the Savitzky-Golay Filter
minLimitWN = 0; % Lower Wavenumber Limit first Multilinear Regression
maxLimitWN = 3500; % Upper Wavenumber Limit first Multilinear Regression
minLimitWN2 = 1500; % Lower Wavenumber Limit second Multilinear Regression
maxLimitWN2 = 3100; % Upper Wavenumber Limit second Multilinear Regression


%% select file(s)

% Choosing the .mat data file
% Criteria: - .mat file
%           - image from WiTec (Project FIVE or similiar)

fBas = 'C:\.....'; % Paste in your file pathway!
[fileName, pathName] = uigetfile([fBas+"\"+'*.mat'], '*.mat',... % opens a
dialogue within the selected folder that have a .mat ending
'MultiSelect', 'on'); % *.mat : show only .mat files; MultiSelect enables to open
multiple .mat files at once

fprintf(" \n Loading raw data \n "); % gives you written output
tic % times how long is takes

fullPath = fullfile(pathName, fileName); % gives you the full path to your data as
output

rawImage.fileName = fileName;
rawImage.fullPath = fullPath;


%% Load File

% As the name implies. Raw data and information will be stored in rawImage.

image = load(fullPath,"-mat"); % the selected folder is loaded
image = struct2cell(image(1,1)); % converts a structure into a cell array
rawImage.Data = image{1,1}.data; % displays the data in the array as an image
rawImage.Name = image{1,1}.name; % extracts name
```

```
rawImage.Date = image{1,1}.date; % extracts date
rawImage.Imagesize = image{1,1}.imagesize; % extracts imagesize
rawImage.Axisscale = image{1,1}.axisscale; % extracts axis scale
rawImage.Imageaxisscale = image{1,1}.imageaxisscale; % extracts image axis scale
imageSize = rawImage.Imagesize;

clear fileName;
clear fullPath;
clear pathName;
clear fBas;


%% Load References

%Loading predefined References and creating a Ref-matrix

load("C:\....."); % add the path to your glass reference
load("C:\....."); % add the path to your PBS reference
load("C:\....."); % add the path to your PDMS reference
load("C:\....."); % add the path to your undeuterated bacteria reference
load("C:\....."); % add the path to your deuterated bacteria reference

rawRef(1,:) = zeros(1600,1);
rawRef(1,:) = glas.data; % glass
rawRef(2,:) = medium.data; % PBS
rawRef(3,:) = pdms.data; % PDMS
rawRef(4,:) = smeggi_undeuteriert.data; % undeuterated bacteria
rawRef(5,:) = smeggi_deuteriert.data; % deuterated bacteria

toc
fprintf(" \n Now cutting raw data \n ");


%% Cutting

% Cutting WN-range to spec. Choose Limits in "Options".

[max, maxIndexWN] = min(abs((image{1,1}.axisscale{2,1}(:))- maxLimitWN));
[min, minIndexWN] = min(abs((image{1,1}.axisscale{2,1}(:))- minLimitWN));
xraw=(rawImage.Axisscale{2,1}(:));
clear image;

data_processing.processingVar.minLimitWN = minLimitWN;
data_processing.processingVar.maxLimitWN = maxLimitWN;
data_processing.processingVar.minIndexWN = minIndexWN;
data_processing.processingVar.maxIndexWN = maxIndexWN;
data_processing.processingVar.min = min;
data_processing.processingVar.max = max;

cutRef = rawRef(:,minIndexWN:maxIndexWN)';
cutData = double(rawImage.Data(:,minIndexWN:maxIndexWN)');


clear min;
clear max;
clear minLimitWN;
clear maxLimitWN;
clear minIndexWN;
clear maxIndexWN;

toc
```

```
fprintf(" \n Already preprocessing data \n ");


%% Data processing

% fist applying a background subtraction, based off Schulz et al. 2012
(https://doi.org/10.1366/11-06550)
% second applying a Savitzky-Golay smoother to remove cosmic ray signals,
% last, normalising the data via min-max to avoid z drift differences.

for n = 1:size(cutData,2)
    [Base, corr_cutData(:,n)] = baseline(cutData(:,n));
    corr_cut_SG_Data(:,n) = sgolayfilt(double(corr_cutData(:,n)), SavGolOrder,
SavGolWindow);
    corr_cut_SG_MinMax_Data(:,n) = (corr_cut_SG_Data(:,n) -
min(corr_cut_SG_Data(:,n))) / (max(corr_cut_SG_Data(:,n)) -
min(corr_cut_SG_Data(:,n)));
    range = max(corr_cut_SG_Data(:,n)) - min(corr_cut_SG_Data(:,n));
if range > 0
    corr_cut_SG_MinMax_Data(:,n) = (corr_cut_SG_Data(:,n) -
min(corr_cut_SG_Data(:,n))) / range;
else
    corr_cut_SG_MinMax_Data(:,n) = zeros(size(corr_cut_SG_Data(:,n)));
end
end

data_processing.Base  = Base;
data_processing.cutData  = cutData;
data_processing.corr_cutData  = corr_cutData;
data_processing.corr_cut_SG_Data  = corr_cut_SG_Data;
data_processing.corr_cut_SG_MinMax_Data  = corr_cut_SG_MinMax_Data;
processedData = corr_cut_SG_MinMax_Data;

clear n;
clear Base;
clear corr_cut_SG_MinMax_Data;
clear corr_cutData;
clear corr_cut_SG_Data;

toc
fprintf(" \n ...preprocessing reference data \n ");


%% Reference processing

% fist applying a background subtraction, based off Schulz et al. 2012
(https://doi.org/10.1366/11-06550)
% second applying a Savitzky-Golay smoother to remove cosmic ray signals,
% last, normalising the data via min-max to avoid z drift differences.

for n = 1:size(cutRef,2)
    [RefBase, corr_cutRef(:,n)] = baseline(cutRef(:,n));
end
corr_cut_SG_Ref = zeros(size(corr_cutRef));
for n = 1:size(corr_cutRef,2)
    corr_cut_SG_Ref(:,n) = sgolayfilt(double(corr_cutRef(:,n)), SavGolOrder,
SavGolWindow);
    corr_cut_SG_MinMax_Ref(:,n) = (corr_cut_SG_Ref(:,n) -
min(corr_cut_SG_Ref(:,n))) / (max(corr_cut_SG_Ref(:,n)) -
min(corr_cut_SG_Ref(:,n)));
end
```

```
data_processing.RefBase  = RefBase;
data_processing.cutRef  = cutRef;
data_processing.corr_cutRef  = corr_cutRef;
data_processing.corr_cut_SG_Ref  = corr_cut_SG_Ref;
data_processing.corr_cut_SG_MinMax_Ref  = corr_cut_SG_MinMax_Ref;

processedRef = corr_cut_SG_MinMax_Ref;

clear n;
clear RefBase;
clear corr_cutRef;
clear corr_cut_SG_Ref;
clear corr_cut_SG_MinMax_Ref;
clear cutData;
clear cutRef;

toc
fprintf( "\n finally calculating multilinear regression \n ");


%% Multilinear regression

% bog-standard multilinear regression
% b (coefficient vector) and r (residual matrix) are stored under regResults

for n = 1:size(processedData,2)

    [b,bint,r] = regress(processedData(:,n),processedRef);
    regResults.b(:,n) = b;
    regResults.bint(n).bint = bint;
    regResults.r(:,n) = r;
end

sum_b = sum(regResults.b(:, :), 1);
b_norm = regResults.b ./ sum_b;
image_deuteriert_norm = reshape(b_norm(5, :), rawImage.Imagesize);
image_undeuteriert_norm = reshape(b_norm(4, :), rawImage.Imagesize);


%% CleanUp

clear n;
clear r;
clear bint;
toc
fprintf(" \n ...time for a coffee! \n ");


%% Visualization of the distribution of Undeuterated bacteria

b_norm = regResults.b ./ sum(regResults.b, 1);

image_smeggi_undeuteriert = reshape(b_norm(4, :), rawImage.Imagesize);
image_smeggi_undeuteriert(image_smeggi_undeuteriert < 0) = 0;

if max(image_smeggi_undeuteriert(:)) > 0
    image_smeggi_undeuteriert = image_smeggi_undeuteriert /
max(image_smeggi_undeuteriert(:));
end
```

```matlab
% Plot
figure;
imagesc(image_smeggi_undeuteriert);
axis image off;
colorbar;
title('\it M. smegmatis');
colormap(parula);

xticks = 1:round(imageSize(2)/5):imageSize(2); % z.B. 5 Ticks auf x-Achse
yticks = 1:round(imageSize(1)/5):imageSize(1); % z.B. 5 Ticks auf y-Achse
set(gca, 'XTick', xticks, 'YTick', yticks);
xlabel('Pixel / Spektrum (Spalten)');
ylabel('Pixel / Spektrum (Zeilen)');


%% Second Multilinear Regression: only for spectra with a positive b-value for
bacteria in the first multilinear regression

rawRef(1,:) = zeros(1600,1);
rawRef(1,:) = glas.data;
rawRef(2,:) = medium.data;
rawRef(3,:) = pdms.data;
rawRef(4,:) = smeggi_undeuteriert.data;
rawRef(5,:) = smeggi_deuteriert.data;

fprintf("\n Starting second multilinear regression on filtered spectra \n");

% Step 1: Identify relevant spectra
positive_indices = find(regResults.b(4, :) > 0);
filtered_pixel_indices = positive_indices;

% Step 2: Define new wavenumber range
[min2, minIndexWN2] = min(abs(rawImage.Axisscale{2,1}(:) - minLimitWN2));
[max2, maxIndexWN2] = min(abs(rawImage.Axisscale{2,1}(:) - maxLimitWN2));

% Step 3: Extract and preprocess selected spectra
selectedSpectra = double(rawImage.Data(positive_indices,
minIndexWN2:maxIndexWN2))';

corr_selected = zeros(size(selectedSpectra));
sg_selected = zeros(size(selectedSpectra));
norm_selected = zeros(size(selectedSpectra));

for n = 1:size(selectedSpectra, 2)
 [~, corr_selected(:,n)] = baseline(selectedSpectra(:,n));
 sg_selected(:,n) = sgolayfilt(double(corr_selected(:,n)), SavGolOrder,
SavGolWindow);
 norm_selected(:,n) = (sg_selected(:,n) - min(sg_selected(:,n))) /
(max(sg_selected(:,n)) - min(sg_selected(:,n)));
end

% Step 4: Extract and preprocess selected references (PDMS, undeuterated bacteria,
deuterated bacteria)
ref_indices = [2, 3, 4, 5]; % PDMS, undeuterated bacteria, deuterated bacteria
selectedRefs = rawRef(ref_indices, minIndexWN2:maxIndexWN2)';

for n = 1:size(selectedRefs, 2)
 [~, corr_ref(:,n)] = baseline(selectedRefs(:,n));
 sg_ref(:,n) = sgolayfilt(double(corr_ref(:,n)), SavGolOrder, SavGolWindow);
 norm_ref(:,n) = (sg_ref(:,n) - min(sg_ref(:,n))) / (max(sg_ref(:,n)) -
min(sg_ref(:,n)));
```

```
end

% Step 5: Multilinear regression
for n = 1:size(norm_selected, 2)
 [b2, ~, r2] = regress(norm_selected(:,n), norm_ref);
 regResults2.b(:,n) = b2;
 regResults2.r(:,n) = r2;
end

fprintf("\n Second multilinear regression complete! \n");


%% Visualization of the b-value ratio distribution of deuterated to undeuterated
bacteria

% Extract b-values from second regression
b_deuterated = regResults2.b(3, :);
b_undeuterated = regResults2.b(2, :);

% Avoid division by zero
ratio = zeros(size(b_deuterated));
valid = b_undeuterated ~= 0;
ratio(valid) = b_deuterated(valid) ./ b_undeuterated(valid);

clip_limit = prctile(ratio, 99);
ratio = min(ratio, clip_limit);

image_ratio = nan(prod(rawImage.Imagesize), 1);
image_ratio(filtered_pixel_indices) = ratio;
image_ratio = reshape(image_ratio, rawImage.Imagesize);

% Plot
figure;
imagesc(image_ratio);
axis image off;
colorbar;
title('Ratio of deuterated to undeuterated {\it M. smegmatis}');
colormap(parula);
```

References

1. Wang, Z.; Pakoulev, A.; Pang, Y.; Dlott, D. D., Vibrational substructure in the OH stretching transition of water and HOD. *The Journal of Physical Chemistry A* **2004,** *108* (42), 9054-9063.
2. Koroleva, O.; Shabunina, L.; Bykov, V., Structure of borosilicate glass according to raman spectroscopy data. *Glass and Ceramics* **2011,** *67* (11-12), 340-342.
3. Konijnendijk, W. L.; Stevels, J., The structure of borosilicate glasses studied by Raman scattering. *Journal of Non-Crystalline Solids* **1976,** *20* (2), 193-224.
4. Cai, D.; Neyer, A.; Kuckuk, R.; Heise, H. M., Raman, mid-infrared, near-infrared and ultraviolet–visible spectroscopy of PDMS silicone rubber for characterization of polymer optical waveguide materials. *Journal of Molecular Structure* **2010,** *976* (1-3), 274-281.
5. Movasaghi, Z.; Rehman, S.; Rehman, I. U., Raman Spectroscopy of Biological Tissues. *Applied Spectroscopy Reviews* **2007,** *42* (5), 493-541.
6. Baron, V. O.; Chen, M.; Clark, S. O.; Williams, A.; Hammond, R. J.; Dholakia, K.; Gillespie, S. H., Label-free optical vibrational spectroscopy to detect the metabolic state of *M. tuberculosis* cells at the site of disease. *Scientific Reports* **2017,** *7* (1), 1-9.
7. De Gelder, J.; De Gussem, K.; Vandenabeele, P.; Moens, L., Reference database of Raman spectra of biological molecules. *Journal of Raman Spectroscopy: An International Journal for Original Work in all Aspects of Raman Spectroscopy, Including Higher Order Processes, and also Brillouin and Rayleigh Scattering* **2007,** *38* (9), 1133-1147.
